# Supplementary material for: Flexible large-area ultrasound arrays for medical applications made using embossed polymer structures
Source: Nat Commun. 2024 Mar 30;15:2802. doi: 10.1038/s41467-024-47074-1 (PMC10981753; doi:10.1038/s41467-024-47074-1)
Supplement: Supplementary file 1 — Supplementary information [file 41467_2024_47074_MOESM1_ESM.docx]

Supplementary information for

**Flexible large-area ultrasound arrays for medical applications made using embossed polymer structures**

Paul L.M.J. van Neer^1^, Laurens C.J.M. Peters^2^, Roy G.F.A. Verbeek^2^, Bart Peeters^2^, Gerard de Haas^2^, Lars Hörchens^1^, Laurent Fillinger^1^, Thijs Schrama^1^, E.J.W. Merks-Swolfs^1^, Kaj Gijsbertse^3^, Anne E.C.M. Saris^4^, Moein Mozaffarzadeh^4^, Jan M. Menssen^4^, Chris L. de Korte^4,5^ , Jan-Laurens P.J. van der Steen^2^, Arno W.F. Volker^1^, Gerwin H. Gelinck^2^

^1^ Acoustics and Sonar, TNO, The Hague, The Netherlands.

^2^ Holst Centre, TNO, High Tech Campus 31, 5656 AE Eindhoven, The Netherlands.

^3^ Human Performance, TNO, Soesterberg, The Netherlands.

^4^ Medical Ultrasound Imaging Center, Department of Medical Imaging, Radboud University Medical Centre, Nijmegen, The Netherlands

^5^ Physics of Fluids Group, Techmed Centre, Twente University, Enschede, the Netherlands

**Contents**

[Supplementary Information Note 1 Mechanical considerations and EUS measurements 4](#_Toc160121125)

[Supplementary Information Note 2 Overview of ultrasound imaging using flexible prototypes 6](#_Toc160121126)

[Supplementary Information Note 3 Stamp stability 9](#_Toc160121127)

[Supplementary Information note 4 Modified KLM model and the frequency dependence of P(VDF-TrFE) model parameters 12](#_Toc160121128)

[Supplementary Information Note 5 Crosstalk 15](#_Toc160121129)

[Supplementary Information Note 6 Scaling to large area 19](#_Toc160121130)

[Supplementary Information Note 7 Photographs of transducer array, its connections and sample holder used in tissue-mimicking phantom measurements 22](#_Toc160121131)

[Supplementary Information Note 8 Blood pressure measurements 24](#_Toc160121132)

[Supplementary References 31](#_Toc160121133)

# Supplementary Information Note 1 Mechanical considerations and EUS measurements

Even though the prototypes presented here are ~0.1 mm thick, they are mechanically robust. The structures have an interfacial normal strength of 15 N/cm^2^ before plastic deformation occurs. Although no endurance testing was performed, the prototypes operated in the lab for more than a year without mechanical or performance degradation. The mechanical flexibility of the bare transducer is effectively determined by the polyimide substrate, bottom P(VDF-TrFE) residual layer and the top laminated P(VDF-TrFE) (**Fig. S1**). This poses an asymmetrical stack, since the lower polyimide layer has a lower elasticity modulus than the P(VDF-TrFE). As a result, the thin top P(VDF-TrFE) laminate will either stretch upon wrapping the foil around a round object (such as an IVUS or EUS probe, see Fig. 1e and S1), or compress when wrapped around a round target object (i.e. patient neck, chest, etc., Fig. 1f). In case of compression, the top laminate even displays small arches in between the pillars on the surface normal to the bending axis. We have observed that the dominant failure mode for the transducer is cracking of the electrode on this top surface. These fractures appear parallel to the bending radius.


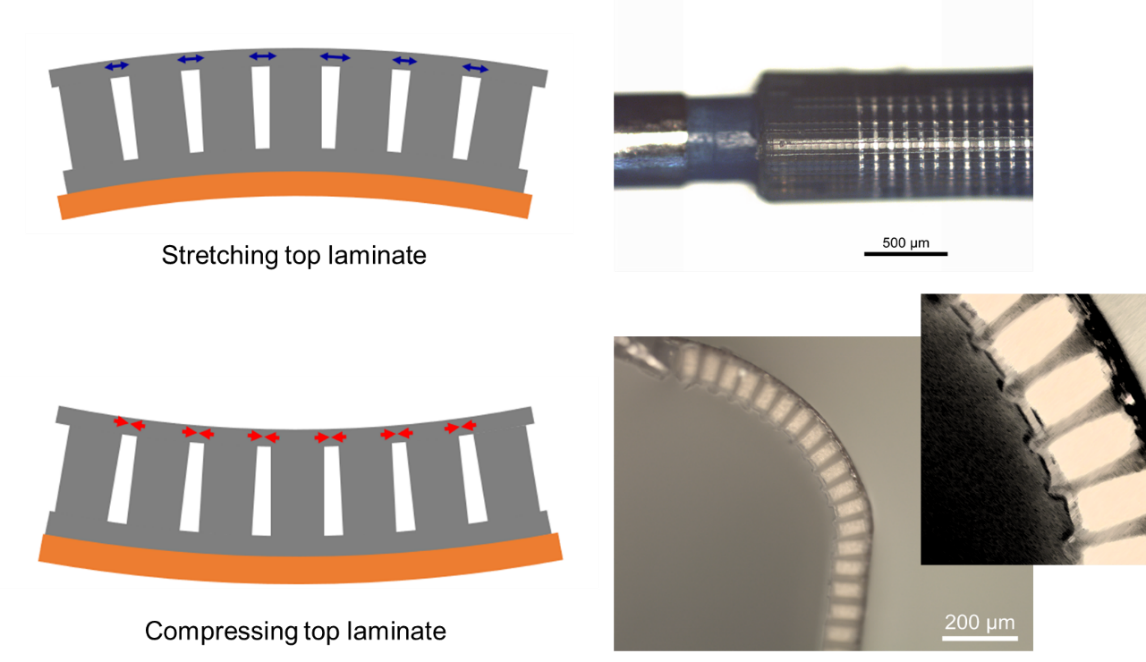


**Figure S1. Mechanical considerations.** Schematic cross section of a PillarWave^TM^ stack with the top laminate layer either stretched or compressed (left top and bottom). Transducer foil wrapped around the inner wire of a PTCA dilatation catheter (Blue Medical Force NC) (right top). Photographs of the transducer cross section while the top laminate is compressed (right bottom). Inset right bottom: magnification of transducer stack under compression of the top laminate.

The mechanical flexibility of the transducer technology is demonstrated by wrapping the 0.1 mm thick PillarWave^TM^ foil around a 6 mm EUS probe and the 0.5 mm inner wire of a PTCA dilatation catheter (Blue Medical Force NC). While the IVUS probe is only fabricated to demonstrate the mechanical aspects of the transducer technology, with the EUS probe we demonstrate that the transducer functionality is still intact upon bending the transducer. To this end, the setup in **Fig. S2** was developed, consisting of a PillarWave^TM^ transducer wrapped around a 6 mm EUS probe using two strips of double-sided adhesive (3M) to provide an air backing, a custom PCB integrated into the probe inner volume containing a transimpedance amplifier and connections to external waveform generator (33250, Agilent Technologies, Loveland, Colorado, USA) and oscilloscope (DSO6032A, Agilent Technologies, Loveland, Colorado, USA).


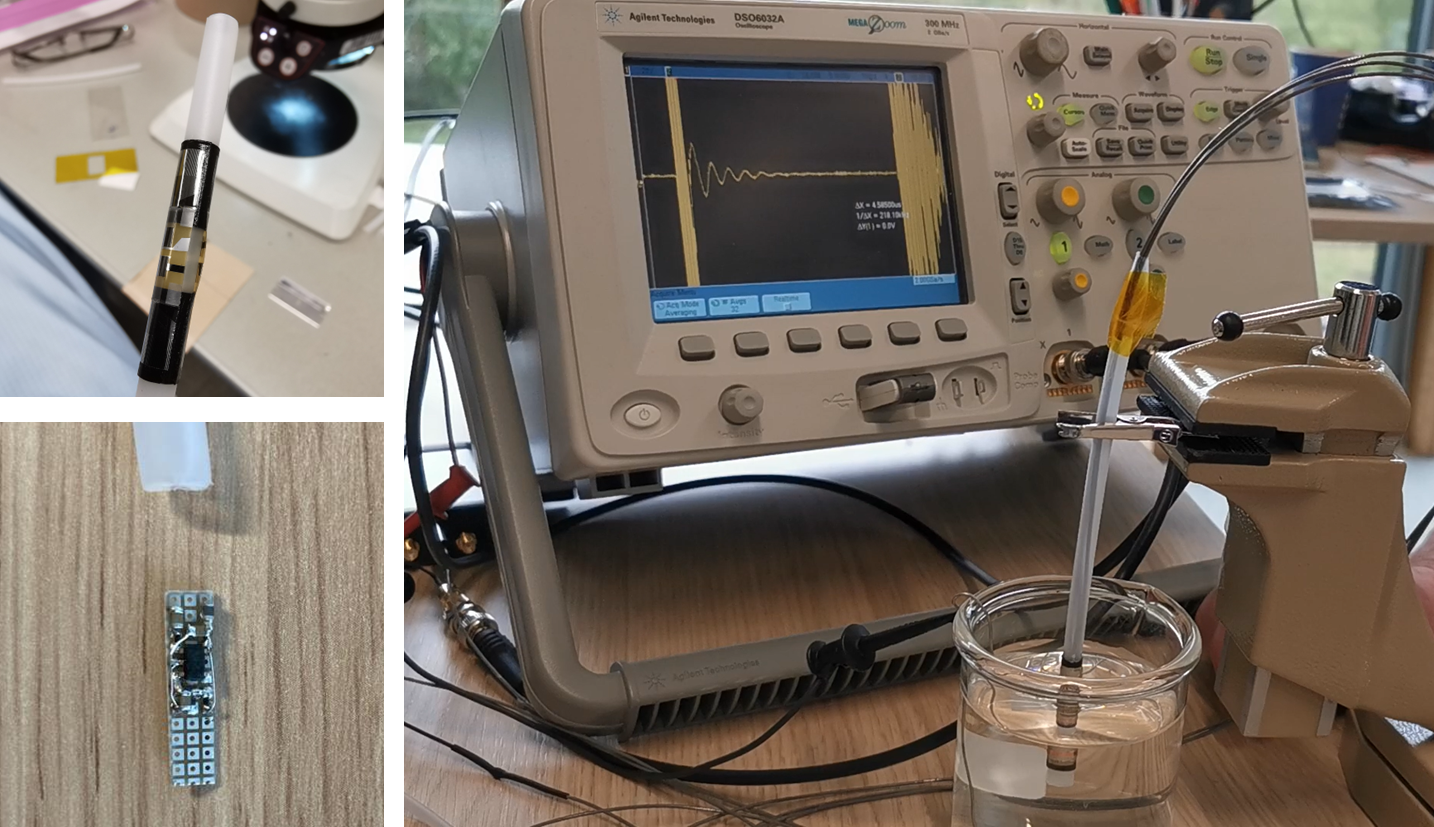


**Figure S2. Experimental setup of EUS measurements.** Photographs of PillarWave^TM^ foil integrated on a 6 mm diameter endoscope probe (Boston Scientific) (left, top), local amplification PCB is integrated into the probe’s inner volume (left, bottom), pulse-echo measurement setup using the EUS probe, waveform generator and oscilloscope (right, large image).

# Supplementary Information Note 2 Overview of ultrasound imaging using flexible prototypes

In 2010 the University of Tokyo created a 2-D arrangement of micromechanical ultrasound transducers on a plastic substrate containing organic thin-film transistors that provided active matrix addressing to the individual ultrasound transducers. ^[[1]](#endnote-1)^ Although the authors of this seminal study were able to image some objects using their sensor, the ultrasound carrier was limited to 40 kHz, preventing any possible application to high-resolution medical imaging, which requires frequencies above 1 MHz.

Several groups demonstrated continuous-wave Doppler ultrasound patches that can acquire blood flow velocities in, for instance, the carotid artery over a long period of ^[[2]](#endnote-2),^^[[3]](#endnote-3)^. The wireless patch typically contains only a few rigid MHz ultrasound elements – one transmit and one receive transducer – and it is created of commercially available ultrasound components.

More recently, Wang et al. reported a stretchable ultrasound patch based on ceramic micromachined rigid ultrasound elements working at 7.5 MHz.^[[4]](#endnote-4)^ Being conformal, the new device overcomes a limitation of today’s rigid and bulky ultrasound devices, which are difficult to use on objects that do not have flat surfaces. Wang et al. successfully demonstrated a 5x4 array that can measure the central blood pressure in deeply embedded arteries. The same group reported a 12x12 phased array operating at 2 MHz with beam forming control^[[5]](#endnote-5)^ and an array of two 5 x 32 elements in an orthogonal configuration sharing the central 5x5 elements^[[6]](#endnote-6)^. The penetration depth was increased to 14 cm beneath the skin due to beam focusing, a higher density of ultrasound elements and by lowering the operating frequency. The technology was used to continuously image deep tissue and monitor for instance cardiac function, which is crucial for detecting cardiac dysfunction and managing cardiovascular diseases in surgical and critically affected patient^5^ and long-term monitoring. ^[[7]](#endnote-7)^

In 2021, Elloian et al. demonstrated a phased 2D ultrasound array containing 16x16 elements on a flexible printed circuit board. The acoustical properties and imaging performance of the FlexArray were characterized using test phantoms and a final prototype was made that could wrap around a human arm to image the humerus.^[[8]](#endnote-8)^ Such wearable ultrasound imagers can increase measurement reproducibility, enable hands-free imaging and offer a solution for (long-term) monitoring applications, without the immediate need of a trained sonographer.

# Supplementary Information Note 3 Stamp stability

The P(VDF-TrFE) layer is structured using a PDMS stamp (*Dow Corning* Sylgard 184). The stamp geometry consist of the inverse of the piezoelectric pillars: an array of square holes in case of square piezoelectric pillars, and a honeycomb structure in case of hexagonal piezoelectric pillars. A cross section of the PMDS stamp belonging to a low aspect ratio square pillar design is shown in **Fig. S3**. To optimize the transducers efficiency, a minimal gap between neighboring pillars is needed, as this increases the effective piezoelectric surface area. This means that the standing wall in on the PDMS stamp should be as thin as possible.

**
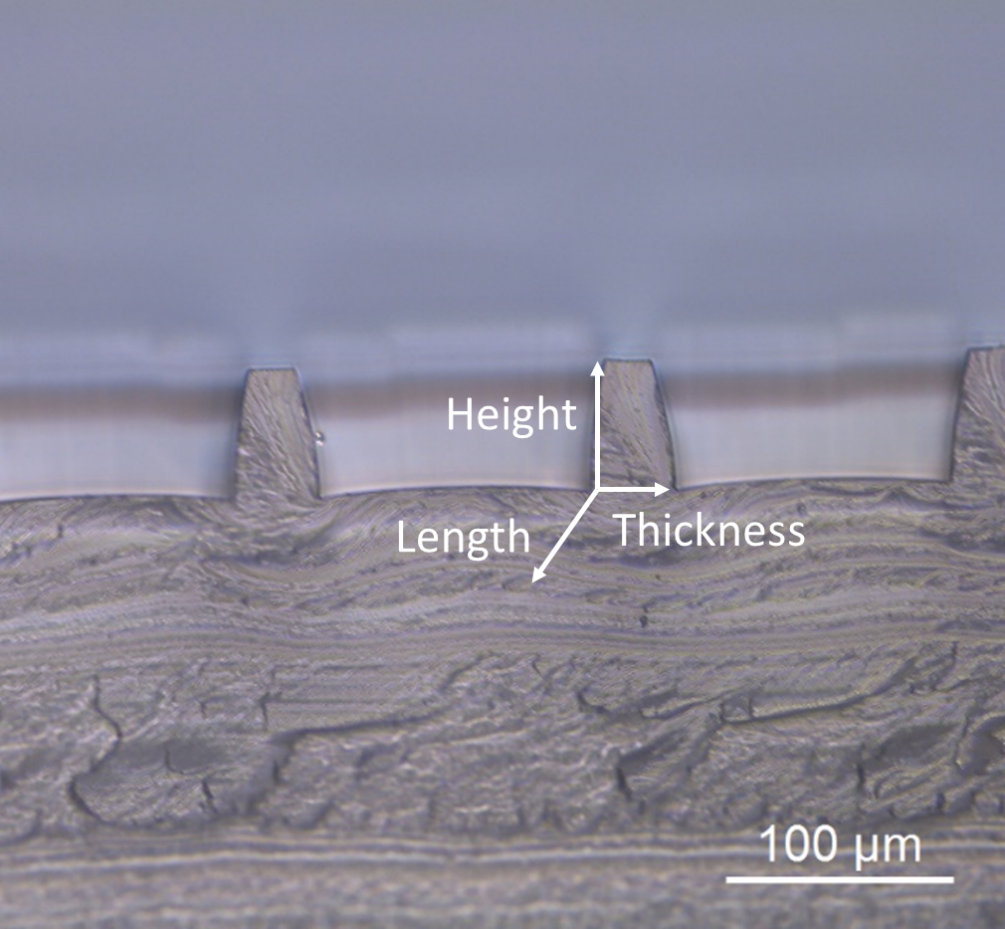
**

**Figure S3. Cross section of a PDMS stamp used for the fabrication of (low aspect ratio) P(VDF-TrFE) pillars**

The stamping fidelity is however limited to the stability (stiffness) of the PMDS walls. When the combination of wall thickness, wall height, and wall length (out-of-plane direction in **Fig. S3**) reaches a threshold value, the standing wall starts to buckle and no longer replicates the intended geometry. For the PDMS stamp shown here, the replicated P(VDF-TrFE) shows buckling of the side walls when performing our embossing process, **Fig. S4.** Focusing on the top of the P(VDF-TrFE) pillar the replication seems successful, however with the microscopic focus on the bottom, curved sides are visible in alternating directions starting from the corner crossings. Since the pillar height (and therefore stamp wall height) is fixed by the application at around 75 µm and the minimum wall thickness is mainly limited by the lithographic fabrication of the SU-8 stamp master, the last degree of freedom entails the wall length between two crossings. We have observed that reducing the wall length between crossings, and therefore effectively the pillar pitch, in the design and processing as described above indeed prevents the wall from buckling. Restricted by this maximum wall length, we are still able to optimize the piezoelectric effective area by change the geometry of the pillars themselves. By taking a hexagonal pattern instead of a square, a higher effective surface is obtained with identical wall thickness and length between crossings, going from 44% to a 64% effective surface.

**
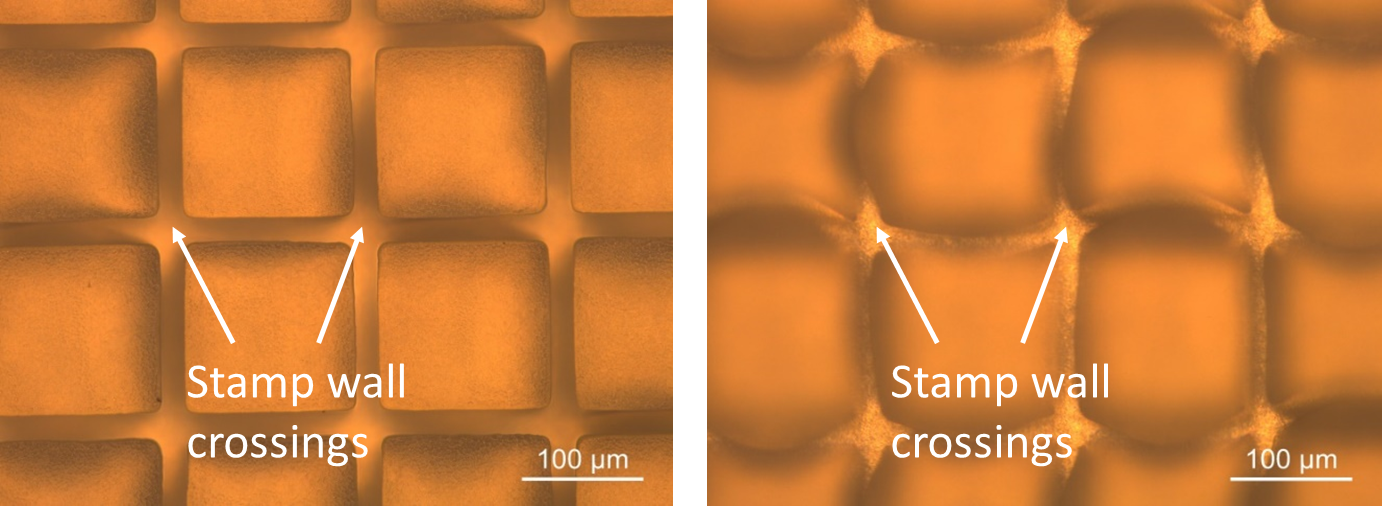
**

**Figure S4. Side wall buckling.** Poor pattern replication of P(VDF-TrFE) pillars is visible as shadows in between the pillars in top focus (left image), and show up as curved walls when the microscop is focused on the bottom part of the pillar structures (right).

# Supplementary Information note 4 Modified KLM model and the frequency dependence of P(VDF-TrFE) model parameters

The regular KLM model^[[9]](#endnote-9)^ is not suitable to describe the behavior of P(VDF-TrFE) transducers. A complication is the strong frequency dependence of the aforementioned P(VDF-TrFE) properties, as indicated by the results of Sherrit et al. (see Fig. 2 in ref ^[[10]](#endnote-10)^). Indeed, many of the formulas commonly used to calculate the piezomaterial constants (e.g., the calculation of the coupling factor based on the resonance and antiresonance frequencies) based on the electrical impedance curve assume high coupling factors and low losses. In this case a more rigorous analysis is required^[[11]](#endnote-11)^. Furthermore, regular Berlincourt meters measure the coupling factors at low frequencies (typically around 1 kHz) and the specification sheets provided by material manufacturers often quote key piezoparameters at similarly low frequencies.

The KLM model is applied most often for thickness mode transducers where the width and length of the piezoelement is much larger compared to the element height. In the case of the PillarWave^TM^ transducer concept, each piezoelement consists of many P(VDF-TrFE) pillars, which each vibrate in a pillar (33) mode. Therefore, applying the 1D KLM model to the aforementioned case is a simplification. The area used in the KLM model (e.g., for calculation of the electrical impedance) is based on the summed area of all poled pillars in a piezoelement.

We have developed a procedure to determine the unknown frequency-dependent parameters by analyzing electrical impedance curves measured in air. The compressional wave speed (*c_p_*), density (*ρ*) and thickness (*d*) of the piezomaterial were measured independently. The initial values for the relative permittivity (*K^S^_33_*) and dielectric (*tan(δ_e_)*) and mechanical losses (*tan(δm)*) were based on the specified values (at 1 kHz) provided by the material supplier. The initial value for the piezoelectric coupling factor (*k_33_*) was calculated from the *d_33_* according to ref. 11. The procedure was as follows:

Obtain a match between the resonance frequencies of the model and experimental data. Although the effective compressional wave speed and thickness of the materials has been measured independently, due to the finite accuracy of the thickness measurement some slight tweaking of the thickness was required.

Determine the frequency independent part of the relative permittivity by fitting the KLM modeled magnitude of the electrical impedance in air at the low MHz range (below the fundamental thickness resonance) to the measured magnitude of the electrical impedance.

Determine the frequency dependency of the relative permittivity by fitting the KLM modeled magnitude of the electrical impedance in air above the fundamental thickness resonance to the measured magnitude of the electrical impedance.

Determine the frequency independent part of the dielectric loss tangent by fitting the low frequency (below the fundamental thickness resonance) KLM modeled phase of the electrical impedance in air to the measured phase of the electrical impedance.

Determine the frequency dependency of the dielectric loss tangent by fitting the slope away from the resonances of the KLM modeled phase of the electrical impedance in air to the measured phase of the electrical impedance.

Determine the piezoelectric coupling factor and mechanical loss tangent by fitting the width and height of the fundamental resonance in the KLM modeled phase spectrum in air to the measured fundamental resonance in the phase spectrum.

Check the obtained parameters by comparing the match between the modeled and measured magnitude and phase of the electrical impedance with air boundary conditions.

The obtained parameters are summarized in the table below.

| **Parameter** | **Unit** | **Value** |
| --- | --- | --- |
| Thickness (*d*) | [µm] | 109 |
| Compressional wave speed (*c_p_*) | [m/s] | 2090 |
| Density (*ρ*) | [kg/m^3^] | 1780 |
| Relative permittivity (*K^S^_33_*) | [-] | 6.5-0.0088$\times frequency(MHz$) |
| Piezoelectric coupling factor (*k_t_*) | [-] | 0.185 |
| Dielectric loss tangent (*tan(δ_e_)*) | [-] | 0.075+0.04 $\times frequency(MHz)$ |
| Mechanical loss tangent (*tan(δm)*) | [-] | 0.125 |

# Supplementary Information Note 5 Crosstalk

In the case of a PillarWave array transducer in contact with tissue, a number of undesired wave modes can occur that effectively cause acoustic crosstalk between neighbouring elements or pillars. (The desired wave mode is the compressional wave emitted into and received from the tissue.)

The first undesired wave modes consist of Lamb waves. Furthermore, a pseudo interface wave traveling along the PillarWave – tissue interface may occur. The dispersion curves for the Lamb waves generally can be predicted from the material properties (e.g. stiffness) and layer thicknesses. However, as the hexagon pillar ‘diameter’ is 40 μm and the kerf between pillars is 10 μm, the waveguide (the flexible array) has a complex shape which is not dominated by the pillars or residual layers alone. Hence, analytical predictions are difficult, as (1) the pillars act as strong scatterers causing mode conversions, and (2) the regular pattern of the pillars produced resonance conditions for the various propagating wave modes.

Instead, an approximate method was used to ensure the pillar spacing was not causing spurious resonances. The top view of the array geometry was 2D Fourier transformed to obtain the normalized spatial frequency spectrum in the (**k_x_**,**k_y_**) domain (Matlab 2023a, Mathworks). Example results for rectangular shaped pillars are shown in Figure 1, where **Figure S5**a shows the spatial frequency spectrum for a pillar kerf of 20 μm and Figure S5b shows the spatial frequency spectrum for a pillar kerf of 0 μm.

Maxima in the spatial frequency spectrum shown in Figure S5 indicate the spatial frequencies where resonances may occur using a particular pillar geometry. Subsequently, the wave speeds of the various wave modes for the frequency range of interest (5-10 MHz) were recalculated to k_x_ and k_y_ ranges using $k_{x}=\frac{2\pi}{\lambda_{x}}$ and $k_{y}=\frac{2\pi}{\lambda_{y}}$, with *λ­_x_* the wavelength in x-direction, expressed as $\lambda_{x}=\frac{c_{x}}{f}$, and *λ­_y_* the wavelength in y-direction, expressed as $\lambda_{y}=\frac{c_{y}}{f}$. For each wave mode this results in a k_x_ and a k_y_ range (essentially a circle or ellipsoid region within Figure S5). If maxima in the spatial frequency spectrum of the pillar geometry are present within the k_x_ – k_y_ range for a particular wave mode, it means a resonant condition may occur. Yet, if the damping of said wave mode is sufficiently high the resonance will be highly damped.

a)
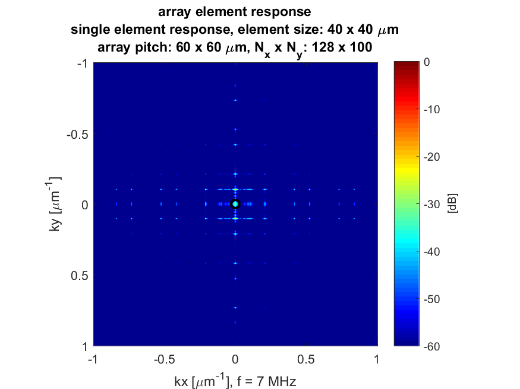


b)
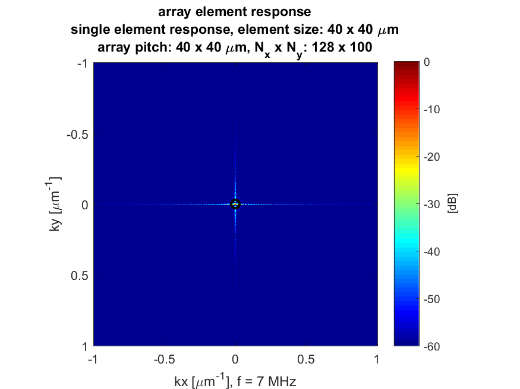


**Figure S5. Array response at 7 MHz as function of element pitch.** a) Pitch of 60 μm. b) Pitch of 40 μm. In b) there is no gap between the elements.

The effective damping of the wave mode can occur due to refraction of energy from the guided wave into the tissue and due to viscous attenuation within the wave guide. We have explored this by implementing a 2D approximation of the PillarWave structure in contact with water in a custom implementation of a 2D viscoelastic Finite Difference model, based on the work of Bohlen (Bohlen, T. (2002). *Parallel 3-D viscoelastic finite difference modelling*. Computers & Geosciences, Vol. 28, pp. 887-889). The viscoelastic losses are modeled for a frequency bandlimited system with a constant Q-factor. We found that when the Q factor is less than 10 the spurious reverberation between the pillars is strongly reduced. As is shown in the supplementary information (Supplementary Information Note 4) the mechanical Q of the single element created using the PillarWave concept was measured to be 8 (tan(δ_m_) = 0.125). (The effective Q will actually be considerably lower due to the contribution of the significant dielectric loss tangent (tan(δ_e_) = 0.075 +0.04 * frequency (MHz)).

So in summary, we have optimized the pillar shape and pillar pitch for the layer structure described in the paper to maximize the effective surface area and to minimize the occurrence of reverberation. Then we used the low impedance of the P(VDF-TrFE) with respect to tissue and the low Q (high attenuative losses) to ensure any reverberation that may occur between pillars is quickly attenuated.

# Supplementary Information Note 6 Scaling to large area


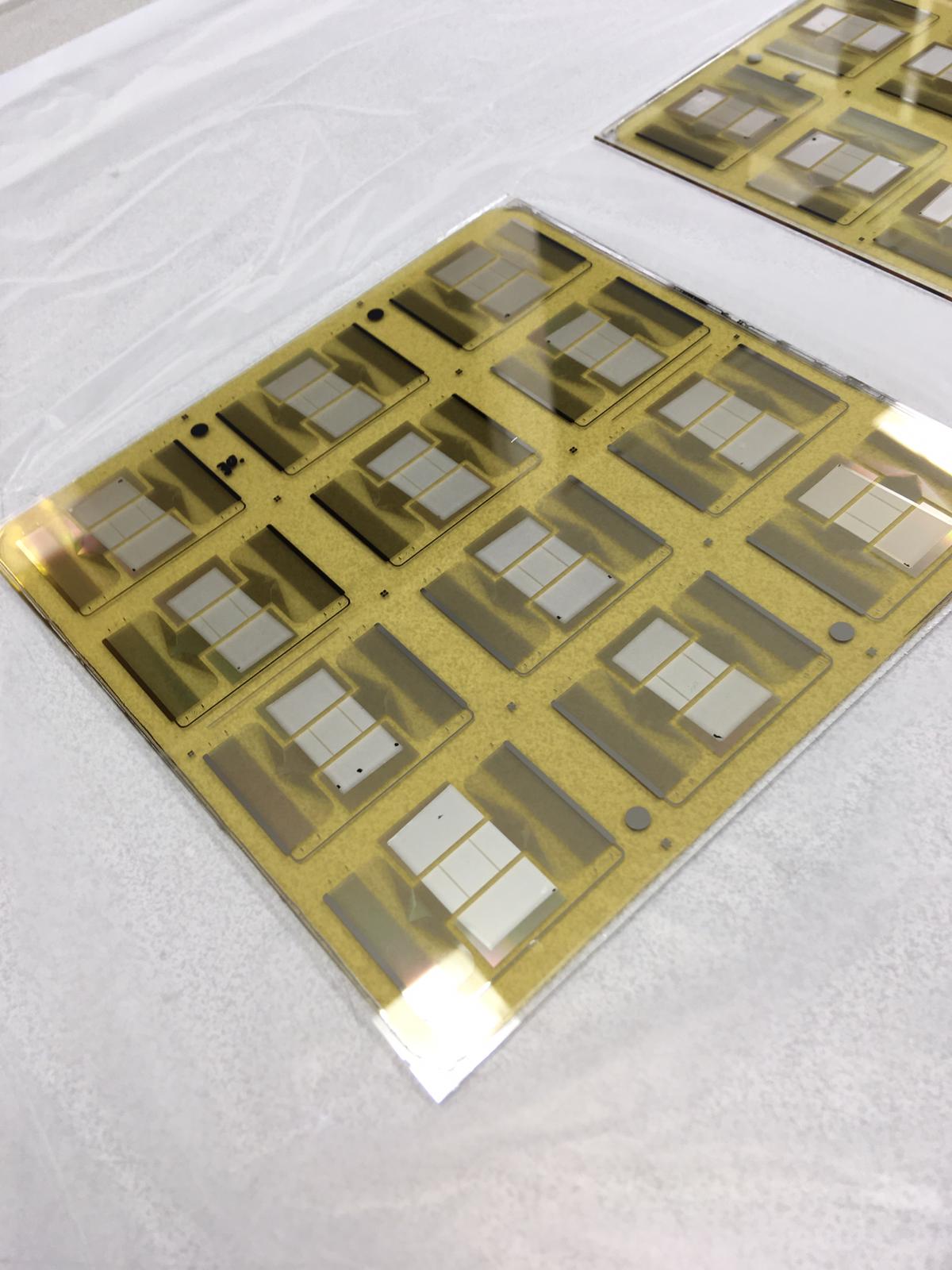


**Figure S6. Photograph of a 15x15 cm^2^ substrate containing 12 transducer arrays.**

# Supplementary Information Note 7 Photographs of transducer array, its connections and sample holder used in tissue-mimicking phantom measurements

**
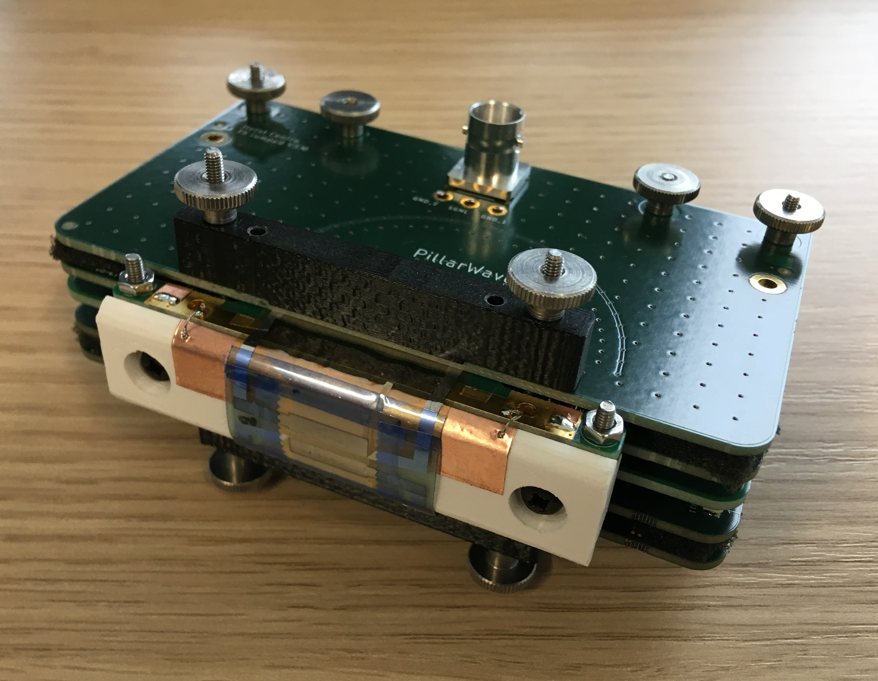
**

**Figure S7. Tissue-mimicking phantom measurements.** Photograph of a 128-ch linear transducer mounted on a (3D printed) sample holder (in white). The fan-out electrodes of the arrays are bent 90° to PCB boards which, among other functions, pre-amplify the receiving signal close to the active elements. The ultrasound array itself was essentially flat during these measurements.

**Figure S8. tissue-mimicking phantom measurements.** Side view of the 128-ch linear transducer array plus PCB board placed on top of the tissue mimicking phantom.

# Supplementary Information Note 8 Blood pressure measurements

In-vitro testing

A carotid artery phantom based on polyvinyl alcohol was created, containing a blood vessel with surrounding tissue. Details of the fabrication process can be found elsewhere.^[[12]](#endnote-12)^ A blood-mimicking fluid was pumped through the vessel using a realistic pulsatile flow waveform at 60 bpm. The pulsatile flow was also monitored using a reference pressure sensor. The setup is shown in the left image of **Fig. S9**, with the array positioned on top of the phantom and coupled to it using ultrasonic gel. The build-up of the phantom was verified with a clinical ultrasound imaging system. The right image of Fig. S9 presents a cross-sectional ultrasound image of the artificial artery, which was used to confirm the diameter and depth below the surface as well as the pulsating movement.


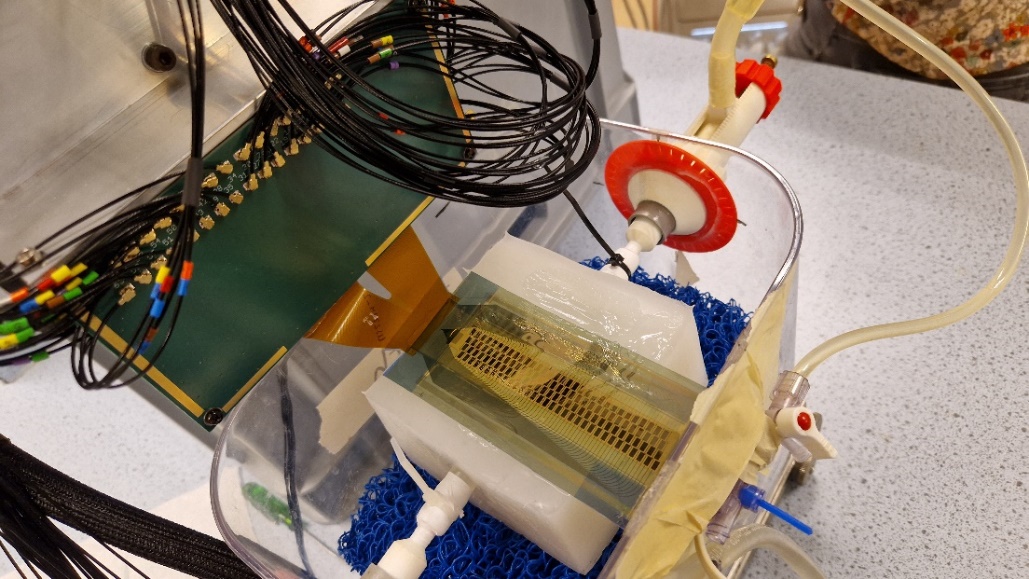

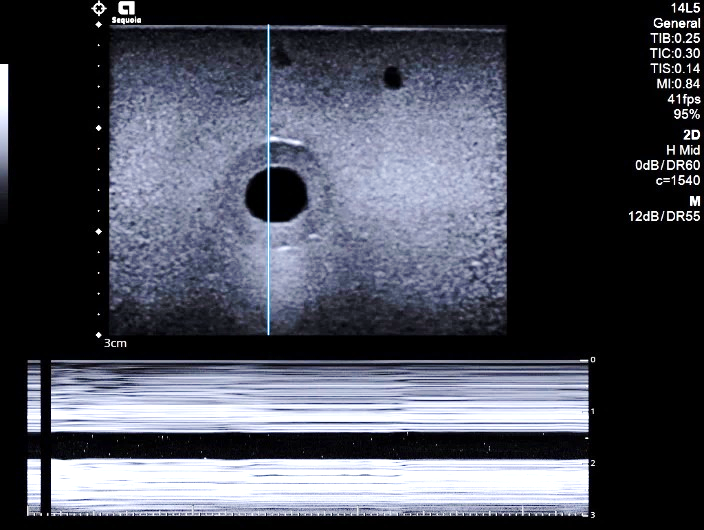


**Figure S9. Blood pressure measurements.** Left image: In vitro setup showing the array on top of a pulsating phantom created to control and mimic a reference blood pressure wave. Right image: Control measurement of the phantom using a medical imaging system to verify construction, diameter and movement of the artificial vessel.

The absolute pressures in the test setup were controlled by restricting the flow using a mechanical clamp on a tube of the flow circuit behind the phantom. Various pulsatile flow waveforms were tested resulting in dynamic pressures of 45/0, 60/20, 100/30, and 130/75 mmHg, respectively. Although some of the former pressures are unrealistically low, the slow build-up was chosen to avoid rupture of the carotid artery phantom and to test our pressure sensing method for multiple dynamic pressure ranges.

The array elements were excited using linear chirps, 20 cycles in length and with a bandwidth of 3-12 MHz. The signals were averaged 8 times. Moreover, interference common to all channels was suppressed by subtracting the mean of all received channels from each individual channel. Out of the four rows of the array, rows 1 and 3 were excited in transmission simultaneously, producing essentially a plane wave propagating into the phantom. Row 2 in between the transmitting elements was read-out in parallel, providing 32 receive channels along the array. Measurements were taken at 50 and 100 frames per second. After initial testing, it was decided that 50 frames per second are sufficient for analyzing the movement of the arterial walls. In all cases, the signal was recorded over a time span of 10 seconds.

The optimal channel (right plot **Fig. S10**) was selected manually from an overview of the receive channels (left plot Fig. S10). In this presentation of the received signals, the position of the walls and the pulsating motion could already be observed. Due to the tube-like construction of the phantom both inner and outer walls of the vessel could be detected in the ultrasonic signals. The array prototype was actually larger than the carotid artery phantom. For this reason, some channels were not positioned above the phantom and did not receive signals. In the selected optimal channel (right plot Fig. S10) the pulsating movement of the vessel was observed in reflections from the anterior and posterior walls.


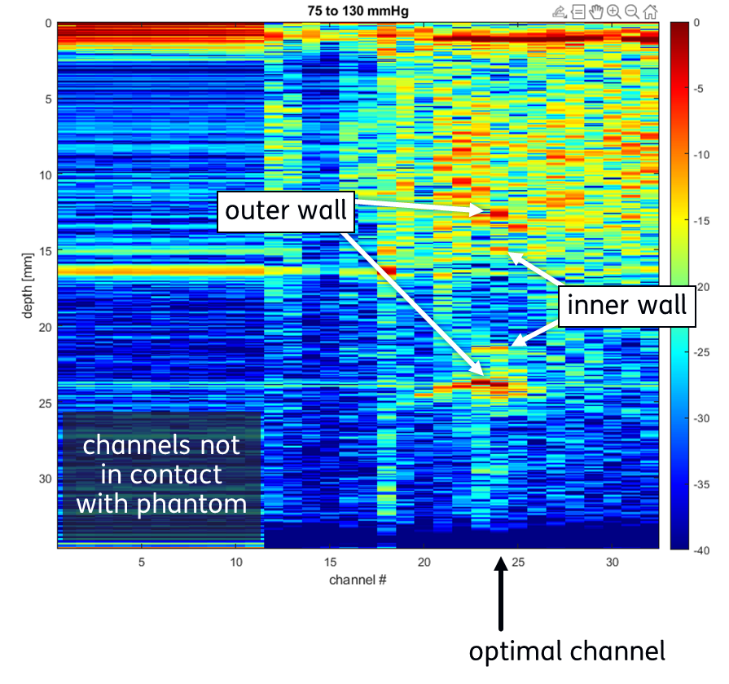

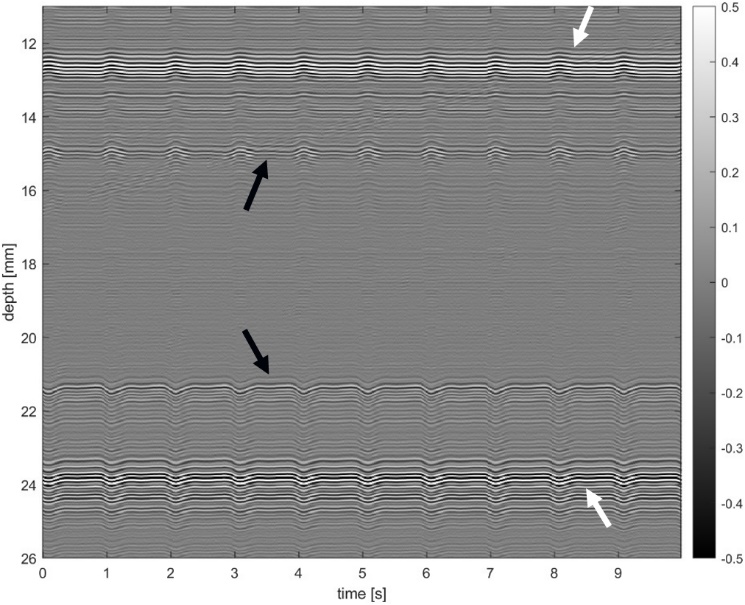


**Figure S10. Blood pressure measurements.** Left plot: signals received from multiple channels (color scale in [dB]) are used to detect the position of the walls of the artificial vessel and select the optimal channel for analysis. Right plot: one channel of the array, showing RF data for 10 seconds at 50 frames per second. Reflections from the inner vessel wall (black arrows) and outer vessel wall (white arrows) are indicated.

The positions of the anterior and posterior wall of the carotid vessel were tracked by calculating the peak of the cross-correlation function of the echoes of the anterior and posterior walls over time with a reference trace. The cross-correlation function was interpolated to enable wall position extraction with sub-sample accuracy. The positions of the anterior and posterior inner wall were used to derive a vessel diameter assuming a circular vessel cross section. The resulting vessel diameters were subsequently converted into blood pressure waveforms using a calibration with the reference pressure sensor. The conversion worked by using the relation between vessel diameter ($A$) and blood pressure ($p$) provided by Wang et al. ^1^:

$p\left( t \right)=p_{d}\cdot e^{\alpha\left( \frac{A\left( t \right)}{A_{d}}-1 \right)}$, (Eq. 1)

with $p_{d}$ the diastolic pressure, $A_{d}$ the diastolic cross-sectional area, and *α* the vessel stiffness coefficient. $A_{d}$ was obtained from the ultrasound data assuming the vessel was circular. Diastolic pressure $p_{d}$ was taken from the reference blood pressure sensor and vessel stiffness $\alpha$ was fitted such that the obtained systolic peak pressure matched with the reference blood pressure.

The derived diameter changes over time for different minimum/maximum pressures are shown in **Fig. S11**. Note that the diastolic diameters for 60/20 mmHg and 100/30 mmHg are almost identical because the simple mechanical clamping applied to the setup changed both flow and pressure at the same time.


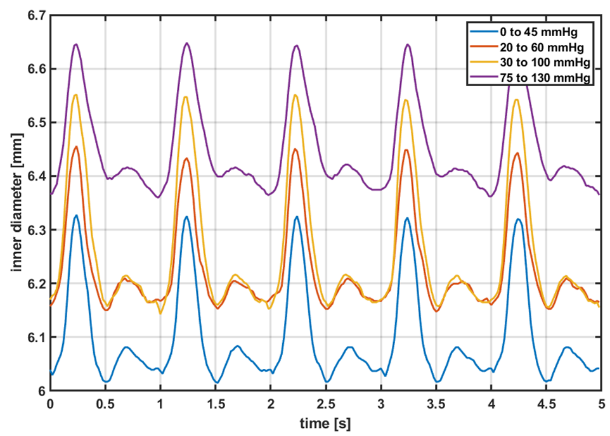


**Figure S11. Blood pressure measurements.** Estimated vessel diameter changes over time measured at several pressures to test the stability of the system.

It was decided to focus on the measurement taken in the medically relevant range of approximately 130 over 75 mmHg. The diastolic pressure was taken from the reference blood pressure sensor. Based on Eq. 1, a fit was made with the vessel rigidity as parameter to be determined. A value for the vessel rigidity was selected to obtain a good match with the maximum pressure of the reference sensor. **Fig. S12** shows the derived blood pressure together with the measurement from the reference pressure sensor. A good correspondence between the measured and reference pressures was observed, with a difference of less than 5%.


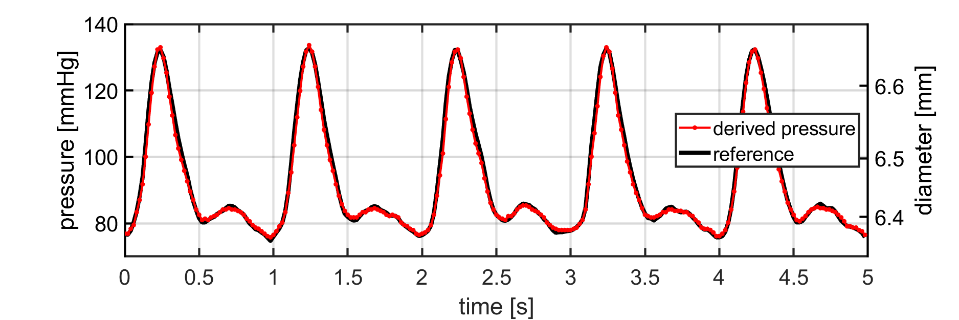


**Figure S12. Blood pressure measurements.** Reference measurement with pressure sensor (black line) and pressure derived from the ultrasonic measurement (red line).

In-vivo testing

In vivo data was obtained on a volunteer by positioning the array on his neck. For this experiment, no reference measurement was made to determine the position of the carotid artery or measure blood pressure over time. The excitation and readout of the array was set up identically to the in vitro case as described in the previous section. Ultrasonic gel was used to provide acoustical coupling between the array and the skin. Slight pressure had to be applied to keep the array in place. Since the array was designed for air backing, a foam material was put on top of the array as an intermediate layer instead of the operator directly pushing with his fingers.

The left image of **Fig. S13** shows an example of recorded receive channels in which the anterior and posterior wall of the carotid artery were identified and well visible in several channels. The pulsation of the artery could be detected in this view. It should be noted that the selection of a channel was easier than in the in vitro case, with the real carotid of the volunteer being at a slightly larger distance from the array than the vessel in the medical phantom. This observation is attributed to the directivity of the individual array elements, with better overlapping coverage at a larger depth. The right image of Fig. S13 shows the data of the array element that was optimally located with respect to the carotid artery. Clear echoes of the vessel walls were observed. The temporal variation, or pulsation, of the inner and outer wall echoes result from the heart beating, which can easily be recognized. Aside from the echoes of the artery wall, there were also other physiological structures leading to additional reflections observed in some of the channels. These are likely caused by muscles or different tissue layers.


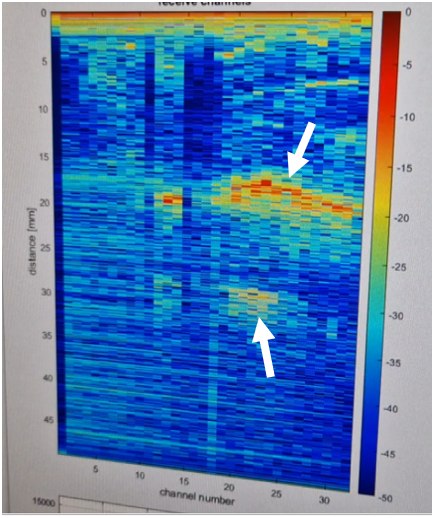

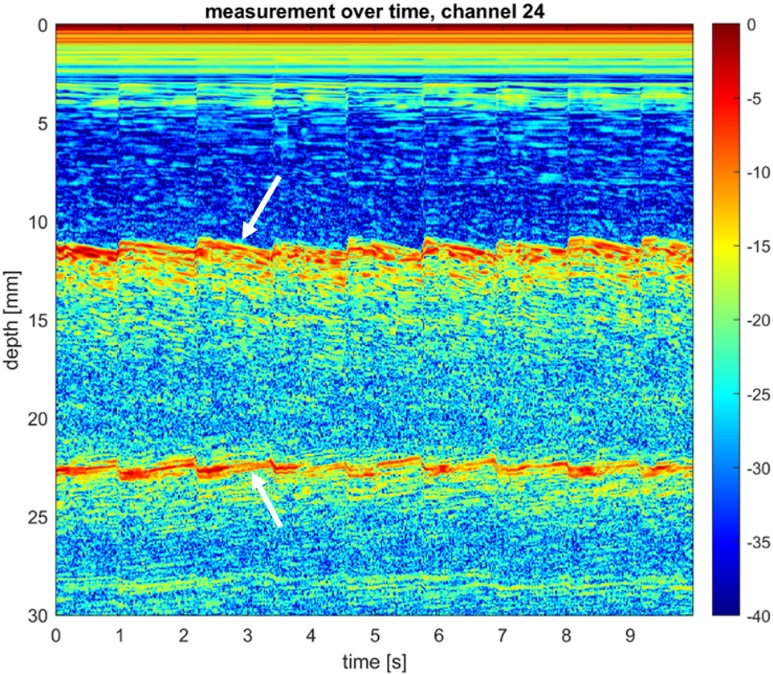


**Figure S13. Blood pressure measurements.** Left image: Screenshot of in-vivo ultrasound measurement show clear anterior and posterior echoes (white arrows) of the volunteers carotid artery. The x-axis shows the number of channels. Right image: carotid wall echoes (white arrows) over time for the selected optimal channel. Please note that the x-axis in this image shows the time.

# Supplementary References

1. Kato et al. Large-Area Flexible Ultrasonic Imaging System With an Organic Transistor Active Matrix. IEEE Trans Electron Dev. 57 995-1002 (2010) [↑](#endnote-ref-1)
2. Ryu et al. Comprehensive pregnancy monitoring with a network of wireless, soft, and flexible sensors in high- and low-resource health settings, *PNAS* **118** e2100466118 (2021) [↑](#endnote-ref-2)
3. Kenny, J.-É. S. *et al.* A novel, hands-free ultrasound patch for continuous monitoring of quantitatively Doppler in the carotid artery. *Sci. Rep.* **11**, 780 (2021). [↑](#endnote-ref-3)
4. Wang. C. *et al.* Monitoring of the central blood pressure waveform via a conformal ultrasonic device. *Nat. Biomed. Engineering.* **2**, 687-695 (2018). [↑](#endnote-ref-4)
5. Wang. C. *et al.* Continuous monitoring of deep-tissue haemodynamics with stretchable ultrasonic phased arrays. *Nat. Biomed. Engineering.* **5**, 749-758 (2021). [↑](#endnote-ref-5)
6. Hu, H. *et al.*. A wearable cardiac ultrasound imager. Nature, **613**, 667–675 (2023). [↑](#endnote-ref-6)
7. Wang, C. *et al.* Bioadhesive ultrasound for long-term continuous imaging of diverse organs. *Science* **377**, 517-523 (2022). [↑](#endnote-ref-7)
8. J. Elloian, J. Jadwiszczak, V. Arslan, J.D. Sherman, D.O. Kessler & K.L. Shepard. Flexible ultrasound transceiver

   array for non‑invasive surface‑conformable imaging enabled by geometric phase correction, *Sci Rep*. **12** 16184 (2022). [↑](#endnote-ref-8)
9. Leedom, D.A. Krimholtz, R. & Matthaei, G.L. Equivalent circuits for transducers having arbitrary even- or odd-symmetry piezoelectric excitation. *IEEE Trans. Sonics Ultrason.* **SU-18** 128–141 (1971). [↑](#endnote-ref-9)
10. Sherrit, S. Haysom, J.E. Wiederick, H.D. Mukherjee, B.K. & Sayer, M., Frequency dispersion and field dependence in the thickness mode material constants of PVDF-TRFE copolymers made by AMP Sensors Ltd *SPIE proceedings (*1998*)* DOI: 10.1117/12.305622 [↑](#endnote-ref-10)
11. Sherrit, S. Mukherjee, B.K. Characterization of Piezoelectric Materials for Transducers, *Dielectric and Ferroelectric Reviews* (2007). [↑](#endnote-ref-11)
12. Fekkes, S. Saris, A. E. C. M. Nillesen, M. M. Menssen, J. Hansen, H. H. G. & de Korte C. L., Simultaneous Vascular Strain and Blood Vector Velocity Imaging Using High-Frequency Versus Conventional-Frequency Plane Wave Ultrasound: A Phantom Studyz, IEEE Transactions on Ultrasonics, Ferroelectrics, and Frequency Control, **65(7)** 1166-1181 (2018). [↑](#endnote-ref-12)
