# Supplementary material for: Flexible large-area ultrasound arrays for medical applications made using embossed polymer structures
Source: Nat Commun. 2024 Mar 30;15:2802. doi: 10.1038/s41467-024-47074-1 (PMC10981753; doi:10.1038/s41467-024-47074-1)
Supplement: Supplementary file 3 — Description of Additional Supplementary Files [file 41467_2024_47074_MOESM3_ESM.pdf]

<u>Description of Additional Supplementary Files</u>

Supplementary Data 1- Comparison between reported ultrasound patches based on number (n) of transducer elements, piezoelectric material, total area of the array, thickness, working frequency, bandwidth, penetration depth, lateral resolution, and axial resolution.
